# Can Priming Multiple Identities Enhance Divergent Thinking for Middle School Students?

**DOI:** 10.3389/fpsyg.2021.704614

**Published:** 2021-10-21

**Authors:** Qian-Nan Ruan, Xin-Wu Ye, Sui-Lin Jia, Jing Liang, Wen-Jing Yan, Yao-Ju Huang

**Affiliations:** ^1^Wenzhou Seventh People’s Hospital, Wenzhou, China; ^2^School of Educational Science, Ludong University, Yantai, China; ^3^College of Teacher Education, Wenzhou University, Wenzhou, China

**Keywords:** creativity, divergent thinking, multiple identities, social priming, scoring method

## Abstract

Previous studies have found that promoting multiple identities can improve children’s creative performance (divergent thinking). The present study employed a priming paradigm to design two experiments and investigate whether promoting a sense of multiple identities in middle school students could enhance their divergent thinking, a key component of creativity. In Experiment 1, 77 junior high school students were divided into multiple identities and physical trait condition groups. They were instructed to think about a child with multiple identities or physical traits. The results showed that there were no differences in divergent thinking (DT) scores between the two groups. In Experiment 2, we modified the priming method by asking participants to think about and write a description of the various identities or physical traits and employed a subjective top-scoring method to make up for shortcomings in the traditional scoring method when applied to originality. The results still showed no significant difference in scores between the identity and physical trait groups. Thus, the results of this study contradict those of previous research, which found that the identity group demonstrated significantly higher scores on a creativity test than did those in the physical trait group. Several potential factors affect this outcome, but it seems that priming to enhance divergent thinking is not particularly effective. Thus, the social priming effect should be pursued with caution regarding both replicability and generalizability.

## Introduction

Creativity is often emphasized as key training content in education, for it has great meaning both for countries and individuals. Half a century ago, Guilford proposed that divergent thinking (DT) is the core of creativity (Guilford, 1967; Sternberg and Grigorenko, 2001). His claim reshaped our views on creativity, and since then DT has held the dominant position in the field of creativity measurement. In particular, DT is assessed according to three aspects: (1) ideational fluency, or the number of ideas an individual has; (2) ideational flexibility, or the number of different conceptual categories used by the individual; and (3) ideational originality, or the statistical infrequency or uniqueness of ideas (Beketayev and Runco, 2016). DT is not synonymous with creativity, but this is a useful quality, enhancing its measurability in relation to creative potential (Runco and Acar, 2012). Accordingly, increasing DT is regarded as beneficial for improving performance on creative tasks.

Creativity or DT is influenced by the complexity of one’s social identification. An individual’s various forms of identification provide openings to different mindsets and angles of thought, which facilitate flexible thinking and help with creative problem solving. Several studies have found that bicultural individuals showed enhanced creativity and professional success, as compared with individuals who identified with only a single culture. This may be explained by their greater levels of integrative complexity, an information processing capacity that involves considering and combining multiple perspectives (Benet-Martínez et al., 2006; Maddux and Galinsky, 2009; Tadmor et al., 2012).

Research has shown that the changing mindsets and feelings related to one’s identity can instantly improve flexible thinking and solving-problem performance. Gaither et al. (2015) observed that people reminded of their multiple racial or social identities generally outperformed the control group in associative and generative creativity, as measured through word tasks (Gaither et al., 2019). The researchers observed that making children aware of their multifaceted identities promoted flexible thinking. Gocłowska and Crisp (2014) argued that possessing two inconsistent identities could foster superior creativity because it allowed for: (a) alternating identities across contexts, (b) integrating elements of distinct (i.e., remote and uncorrelated) identities and, having formed cognitive and emotional links with a new group, a (c) broadening of self-definition. It is meaningful to verify such an observation and better understand the mechanism in operation because doing so will provide us with a key to understanding and fostering creativity and enhancing problem-solving performance.

One convenient method to make people experience feelings related to various identities is priming. Priming refers to providing environmental stimuli that may affect a subject’s responses by activating mental constructs without their conscious realization (Bargh and Chartrand, 2000). In social psychology, researchers call this social or behavioral priming to differentiate it from semantic priming, which refers to the observation that a response from a target (e.g., a dog) is faster when it is preceded by a semantically related prime. Behavioral priming is important in psychological theory because it provide evidence about the influence of automatic or unconscious processes on behavior (Payne et al., 2016). As previous research has shown, the priming of multiple identities seems to improve creativity (flexible thinking). In the present study, we plan to verify the such a priming effect.

With regards to creativity assessments, DT tests are a top priority. Though the validity and reliability of such tests are the subject of much debate, they are still supported by scholars and continue to be popular in research and practice (Runco and Acar, 2012). DT tests are mostly comprised of open questions, requiring subjects to list as many answers as possible, according to the requirements of the question. For example, in one study, participants were asked to write down as many different uses for objects as possible in 2 min (Hass, 2015). Among the DT tests available, the most frequently used include Guilford’s Structure of the Intellect (SOI) (Guilford, 1967), the Torrance’s Test of Creative Thinking (TTCT) (Torrance, 1972), and less commonly, the Wallach-Kogan test.

This study conceptually replicates Gaither’s 2019 research, in which it was observed that making children aware of their multifaceted identities promoted flexible thinking. In this study, we focused on junior middle school students who demonstrated high self-awareness and were asked to solve a problem related to self-identity. Participants of this age have expanded social interactions and a solid understanding of their various social roles (Barenboim, 1981; Burnett and Blakemore, 2009). In addition to the social development of the early adolescents, the schools try to promote the development of creativity at this stage, and middle school students have more time and are more malleable than high school students and adults. By reason, it was assumed that such participants would display a significant effect from multi-identity priming on their creativity or flexible thinking. In Experiment 1, we hypothesized that students primed regarding their multiple identities would offer numerous perspectives, and thus would outperform on DT tests those who were primed regarding physical traits.

## Experiment 1

### Participants

Seventy-seven Chinese students in their first year of middle school (aged 13–14) took part in an experiment. These students were selected from two parallel classes in the same grade, with one class in multiple identities condition (39 participants, 19 females) and the other in physical traits condition (38 participants, 18 females). Neither group of students had taken part in a similar type of experiment before.

### Materials

All participants were presented with instructions that matched their gender. The subjects were guided to recognize multiple identities or physical traits. In the multiple identities condition, participants were led to identify eight identities or physical traits, and experience what it was like to have them all. For example, “Look at this girl! She is a reader, and she is also a friend. Are you a reader? Are you also a friend?” The physical traits instructions were identical, except participants were told that they had eight physical attributes. For example, “Look at this girl! She has two feet and a mouth. Do you have two feet? Do you have a mouth?” After they read the instructions and indicated that they understood, they were asked to sign their name on the instruction sheet. Then, they were asked to recall and write down the eight identities/physical traits on a separate sheet of paper (see Supplementary Material 1).

### Procedure

This experiment was a one-factor between-subjects design. The independent variable of priming condition had two levels: multiple identities and multiple physical traits. The dependent variable was their score on the DT test extracted from the Wallach-Kogan test (Cropley and Maslany, 1969). We selected three items from three sections: Uses, Similarities, and Pattern Meaning. The Kuder-Richardson reliability coefficients on the original test were 0.82, 0.86, and 0.87, respectively. Thus, the DT test in this study consisted of three sections with three items each.

First, the researcher distributed the priming materials (see Supplementary Material 1) to the multiple identities and physical traits groups, asked the subjects to read through the materials on page 1, and try to feel the identities or physical traits listed. Then, the subjects signed their names on page 2, and tried to recall the identities or physical traits and list them. Next, the experimenter distributed a DT test. The time limit was 5 min for each section (three items each), for a total of 15 min. The experimenter encouraged the students to write as many answers as possible (see Supplementary Material 2). They were not allowed to move to the next section until time was up for the first section.

### Data Analysis

#### Scoring the Tests

One participant was removed from the analysis because they did not complete the test. Answers from 76 participants were input into a computer and scored according to three DT dimensions: fluency, flexibility, and originality. Because manual scoring of DT tests is very time-consuming and laborious, researchers have developed an automatic computer-based processing method for word classification and data analysis (Beketayev and Runco, 2016). Subsequent researchers developed a Chinese version of the computerized scoring system (Shen and Shao, 2019). The Kendall coefficients for the samples were 0.860 for fluency, 0.836 for flexibility, and 0.627 for originality (see Supplementary Material 3 for details).

#### Removing Extremes

The data generally followed a normal distribution, with some extremes. For example, most students wrote down fewer than 10 answers for each question, but one listed 18 answers. Extreme values always need to be dealt with because they can significantly impact the average. We calculated the standard deviation of the scores and defined the extreme values as those with standard deviations less than –2.5 or greater than 2.5 (less than 5% of the total data). However, this extreme value was not a mistake and it would not have been suitable to directly eliminate it or replace it with an average. Therefore, the SD of the score outside of the threshold (SD ±2.5) was replaced with the threshold value.

#### Merging the Data

The scores for the fluency, flexibility, and originality sections were averaged to obtain the overall scores for each. Then, the overall fluency, flexibility, and originality scores were averaged to serve as the DT score for each subject.

### Results

An independent samples *t*-test was used to compare the differences in fluency, flexibility, originality, and average scores for the multiple identities and physical traits conditions. The *p* values for these dimensions were close to but greater than 0.05, meaning there may have been marginally significant differences in higher scores for the physical traits rather than the multiple identities condition (see Table 1).

**TABLE 1:** Divergent thinking (DT) scores for multiple identities and physical traits conditions (*N* = 76).

**Dimension**	**Multiple identities**		**Physical traits**		***t***	***p***
	***M***	***SD***	***M***	***SD***		
Fluency	4.523	1.597	5.237	1.669	–1.962	0.054
Flexibility	4.088	1.240	4.646	1.338	–1.888	0.063
Originality	6.015	2.173	6.919	2.395	–1.722	0.089
Average	4.875	1.625	5.600	1.784	–1.852	0.068

We hypothesized that the multiple identities condition would show significantly higher scores for all three DT dimensions. However, the results did not support the hypothesis. Actually, the multiple identities condition score was marginally lower than that of the physical traits condition. This may have been because the multiple identities priming in Experiment 1 did not produce the desired effects. The primed identities/physical traits were already presented in the text and the students could simply recall these words, where they didn’t genuinely feel these identities. We tried to modify the priming approach in Experiment 2 by asking the students to write down the identities/physical traits by themselves, expecting that such an operation would make them more fully aware of their own identities and have better priming effects. In addition, we used subjective top-scoring to score originality in Experiment 2, considering the drawbacks of the traditional approach to originality scoring and the low reliability of originality in the computerized scoring system.

## Experiment 2

Experiment 2 had a similar design to Experiment 1, but each participant was asked to think by themselves and write about the identities/physical traits. In addition, a subjective top-scoring method was used to score originality. Traditional scoring on DT tests suffers from a high correlation between fluency and originality, meaning that more writing leads to higher scores for originality. Scholars have proposed a subjective top-scoring method, where participants are asked to select a number of their most creative ideas for later creativity ratings, avoiding problems such as not confusing originality with fluency and not affected by large sample sizes. Silvia et al. (2008) considered 2 or 3 raters is satisfactory for reliability. In Experiment 2, each participant was asked to circle their three most “creative” answers. Two raters then rated the circled answers on a scale of 1–5, ranging from “not at all creative” to “very creative.” Unusual, distinct, and intelligent (Wilson et al., 1953) were used as scoring criteria. A detailed description is published in the appendix of Silvia et al. (2008). To increase inter-rater agreement, scoring guidelines adapted from Silvia et al. (2008) were learned by the raters. We hypothesized that students primed by multiple identities would score higher on the DT test than those who were primed with physical traits.

### Participants

Eighty-four students (ages 13 and 14) in their first year at a Chinese middle school took part in the experiment, with 42 in the multiple identities and 42 in the physical traits conditions. The groups of students from Experiments 1 and 2 were different. None of the students had taken part in a similar type of experiment before that day.

### Materials

Instead of recalling the identities/physical traits from instructions (as in Experiment 1), the participants in Experiment 2 were required to think about and write down answers on their own. They were encouraged to write as many as possible (see Supplementary Materials). It was expected that such an operation would enhance the priming effect beyond what was seen in Experiment 1.

### Procedure

The procedure was similar to that of Experiment 1, except that participants were asked to circle their three most creative answers to each question.

### Data Analysis

The computerized scoring was similar to what occurred in Experiment 1. On relatively simple tasks such as rating DT tests, novice raters can often do well (Benedek et al., 2013). Two college students were asked to rate the originality of the circled answers and obtain an average score for each. Raters were not involved in the experiment and did not know its purpose. The raters rated the answers to each question on a scale of 1–5, ranging from “not at all creative” to “very creative.”

### Results

An independent samples *t*-test was used to compare the differences in fluency, flexibility, originality, and average scores for the two conditions. Since we used subjective top-scoring for originality, we compared the differences between the two conditions in Originality-S and the corresponding Average-S. The *p* values for these dimensions were much greater than 0.05, indicating that there was no difference between the two conditions (see Table 2).

**TABLE 2:** Divergent thinking test scores for the multiple identities and physical trait conditions (*N* = 76).

**Dimension**	**Multiple identities**		**Physical traits**		***t***
	***M***	***SD***	***M***	***SD***	
Fluency	4.886	1.342	4.603	1.312	0.977
Flexibility	4.474	1.012	4.169	1.275	1.211
Originality	7.005	1.965	6.124	2.300	1.887
Originality-S^*^	6.413	1.082	6.405	1.149	0.033
Average	5.455	1.367	4.966	1.574	1.521
Average-S^*^	5.258	0.945	5.059	0.975	0.947

*Indicates originality dimensions scored by subjective top-scoring, and thus being represented by different averages.

## General Discussion

The present study found that priming students with multiple identities yielded no significantly higher scores on DT tests than did the control condition. The logic and design of Experiment 1 were based on Experiment 1 of a previous study (Gaither et al., 2019), but our results were dissimilar from theirs. In Experiment 2, we modified the priming approach and used a subjective top-scoring method, but still failed to see the effectiveness of priming multiple identities on improving DT performance.

The present study is a conceptual replica of Gaither et al. (2019). However, there were several differences, including the participants, materials, and procedure. The participants in Gaither et al. (2019) were elementary students in lower grades, while in the present study they were students in their first year of middle school. Students at such an age have better social interaction and self-identity development, which could learn more roles and understand the differences between self and others. Due to the age difference, the present study employed tasks more suitable for older students. The materials in Gaither et al. (2019) included functional fixedness, multiple uses, and social categorization tasks, while the present study employed multiple uses, similarities, and pattern meaning tasks commonly found in the Guilford’s SOI, TTCT, and (the less commonly used) Wallach-Kogan tests. There was some overlapping of tasks and several differences, but all required flexible thinking. Therefore, though there are some differences between the present and previous experimental designs, they are basically the same, and students in adolescence are supposed to show a more significant effect. However, the priming effect was not observed.

Since the results of Experiment 1 were not significant, we made some adjustments. Each participant was asked to think about and write down the identities/physical traits on their own. This was expected to get them more involved in feeling the multiple identities, but it made no difference. Moreover, the subjective top-scoring method was used to score originality, in order to avoid the “bad” scoring by computerized scoring system (i.e., the traditional method). However, there was no difference between the subjective top-scoring and traditional scoring in terms of the results for originality.

One likely explanation is that priming may not always work, or may not be particularly robust. This is not surprising because social (rather than semantic) priming is still a topic of debate. Some classic experiments in this area were found not to be replicable. For example, Harris et al. (2013) conducted two experiments and found achievement priming did not improve participants’ performance; thus, the researchers were unable to replicate a previous study (i.e., Bargh et al., 2001). In another study, Shanks et al. (2013) conducted nine experiments and none showed that “intelligent priming” affected performance on a subsequent test of general knowledge (Dijksterhuis and Van Knippenberg, 1998).

Payne et al. (2016) believed that the absence of a social priming effect was caused by problems with the experiment design (as well as other aspects), because social priming studies usually have an inter-subject design and there is only one trial. Also, after an operation begins, it takes some time for the task be completed. Such a design may cause the priming effect to be less significant and not consistently affect subsequent operations. In contrast, semantic priming generally occurs within the subject, there are many trials, and the task is carried out immediately after presentation of the priming trial. Thus, the semantic priming effect is more directly applied to the subsequent task. Thus, the authors designed a social priming experiment using semantic priming as a reference. They obtained consistent results in six experiments. In the present study, as Payne et al. asserted, priming was followed not by just by a small task, but a rather long DT test administered after priming. Thus, the priming effect was very small and we used an inter-subject test. This may explain why there was no effect in the present study. However, our work supports the criticism that the classic social priming paradigm is not robust or even replicable.

Similarly, there have been many studies exploring whether priming can change cheating behavior. However, our previous experiments could not find a similar priming effect in practical situations neither (Wu et al., 2020). At the very least, these findings suggest that the effect of classic social priming is small, so the results must be carefully verified before being applied.

## Data Availability Statement

The raw data supporting the conclusion of this article will be made available by the authors, without undue reservation.

## Ethics Statement

The studies involving human participants were reviewed and approved by IRB in Wenzhou University. Written informed consent from the participants’ legal guardian/next of kin was not required to participate in this study in accordance with the national legislation and the institutional requirements.

## Author Contributions

Q-NR and JL conceived and designed the experiments. X-WY and S-LJ performed the experiments. Q-NR, Y-JH, JL, and W-JY analyzed the data and wrote and revised the manuscript. All authors contributed to the article and approved the submitted version.

## Conflict of Interest

The authors declare that the research was conducted in the absence of any commercial or financial relationships that could be construed as a potential conflict of interest.

## Publisher’s Note

All claims expressed in this article are solely those of the authors and do not necessarily represent those of their affiliated organizations, or those of the publisher, the editors and the reviewers. Any product that may be evaluated in this article, or claim that may be made by its manufacturer, is not guaranteed or endorsed by the publisher.
