# Supplementary material for: Can Priming Multiple Identities Enhance Divergent Thinking for Middle School Students?
Source: Front Psychol. 2021 Oct 21;12:704614. doi: 10.3389/fpsyg.2021.704614 (PMC8566743; doi:10.3389/fpsyg.2021.704614)
Supplement: Supplementary file 1 [file Data_Sheet_1.PDF]

Supplementary Materials 1

Priming materials used in Exp. 1

The following two pages were given to male participants in the multiple identities condition.

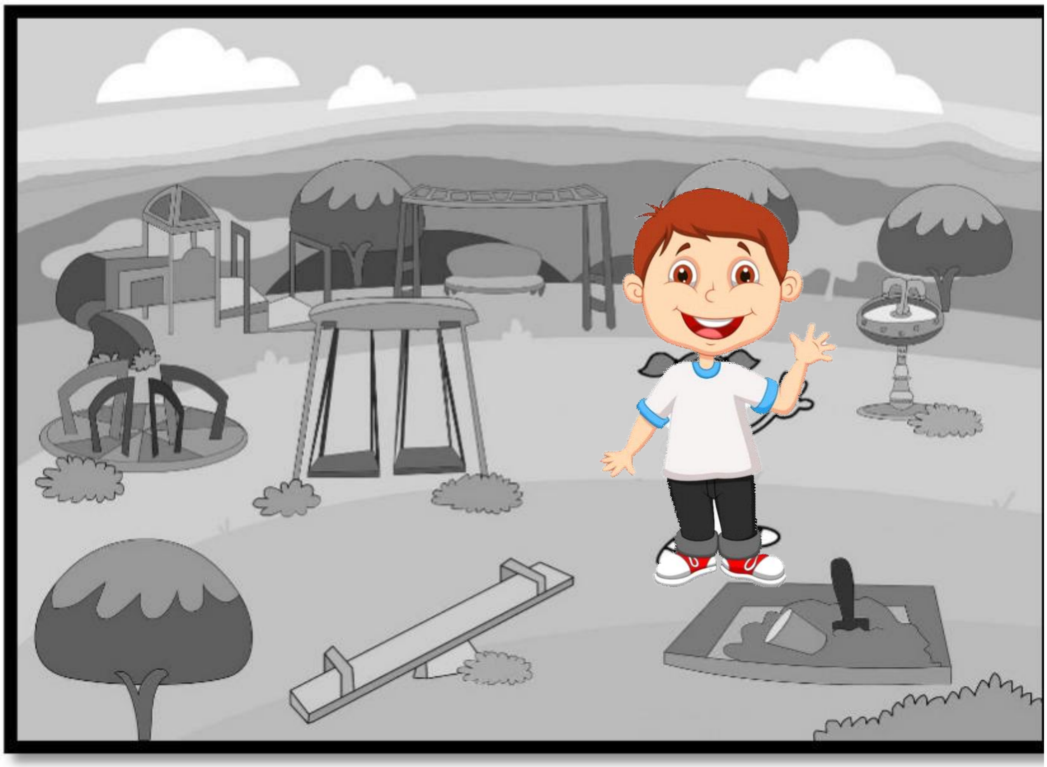

Hello! You're going to play some games today!

Look at this boy. He's in the park! He has two eyes and two ears. Do you have two eyes? Do you have two ears? He also has a mouth and teeth. Do you have a mouth? Do you have teeth? He has a nose and arms. Do you have a nose? Do you have arms? He has legs and feet. Do you have a pair of legs? Do you have feet, too? It's great that you have so many body parts! Please write your name on the line next to the picture of the boy below; this picture represents you. We were talking about the little boy's body parts, and you have a lot of body parts just like he does. Now remember the parts of your body we talked about and write them down next to the little boy who represents you.

Instructions: Recall the parts of your body we talked about and write them down next to the little boy who represents you.

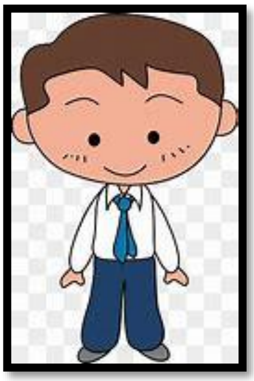

Your name: _______________

1. ____________
2. ____________
3. ____________
4. ____________
5. ____________
6. ____________
7. ____________
8. ____________

Priming materials used in Exp. 1

The following two pages were for the female participants in the physical traits condition.

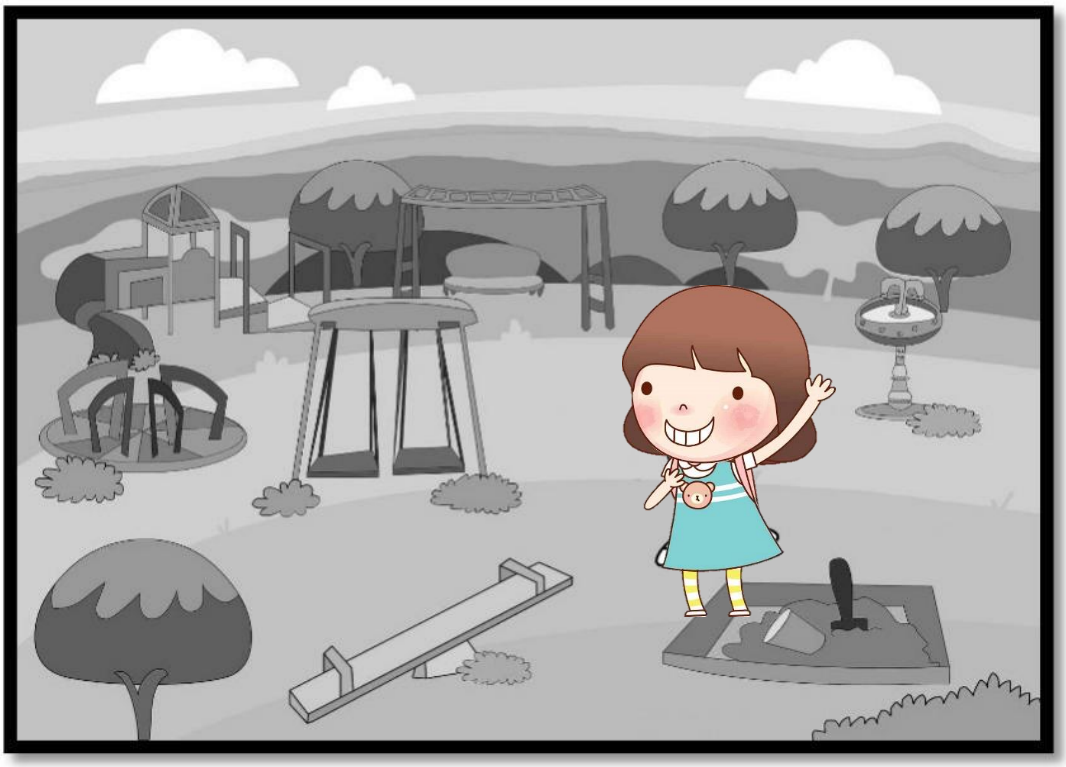

Hello! You're going to play some games today!

Look at this girl. She's in the park! She has two eyes and two ears. Do you have two eyes? Do you have two ears? The girl also has a mouth and teeth. Do you have a mouth? Do you have teeth? She has a nose and arms. Do you have a nose? Do you have arms? She has legs and feet. Do you have a pair of legs? Do you have feet, too? It's great that you have so many body parts! Please write your name on the next line. The child in the picture represents you. We were talking about the little girl's body parts, and you have a lot of body parts just like her. Now, remember the parts of your body we talked about and write them down next to the picture of the little girl who represents you.

Instructions: Recall the parts of your body we talked about and write them down next to the little girl who represents you.

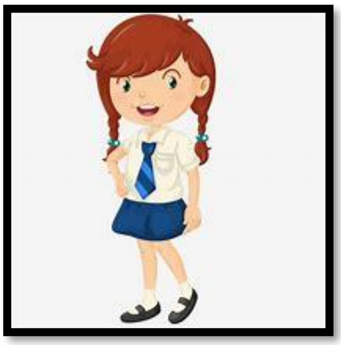

Your name: _______________

1. ____________
2. ____________
3. ____________
4. ____________
5. ____________
6. ____________
7. ____________
8. ____________

Supplementary Materials 2

The following sheets were used by students to list identities or physical traits in Exp. 2.

Instructions:

Everyone has many identities, such as being Chinese, a student, sports participant, class leader, and so on. Write at least eight of your identity roles. [*For the multiple identities condition*]

Everyone has many body parts, such as hair, toes, etc. Write at least eight parts of your body. [*For the physical traits condition*]

Name ______

1. ___________
2. ___________
3. ___________
4. ___________
5. ___________
6. ___________
7. ___________
8. ___________
9. ___________
10. ___________

Supplementary Materials 3
Methods of word classification in the text and creativity assessments

Part A: Steps for word classification

(1) Read the text line-by-line and divide it in a regular manner to obtain words and expressions.
(2) Words obtained in (1) should be further segmented by jieba, simple words obtained, and the word frequency for each simple word counted.
(3) Set parameters for classification, K[*k*，*limit*], where *k* indicates the frequency and *limit* indicates the upper limit of the frequency. The *limit* words with frequencies higher than k should be selected as candidate topics. Then, determine the candidate topics to which the simple words belong. Make a preliminary classification and set M categories, where M $\leqslant$ *limit*.
(4) Choose the word with the highest frequency in each category to serve as the topic of that category.
(5) Go through all the words in each category to determine whether they belong to the topic of that category. If yes, put them into the category; if not, classify them into low-frequency word sets.
(6) Low-frequency word sets should be further divided by the Word2Vec.model.
(7) Give the results.

Part B: Steps for the creativity assessments

(1) Classify the words from the text as in Part A.
(2) Calculate the fluency, flexibility, and originality, based on previous word classifications, and obtain the results for each participant. Fluency refers to the total number of words from all categories (1 point for each correct answer). Flexibility is the number of categories in the text (1 point for each category). Originality is the frequency of a word among all participant answers (0 points if the answer is written by more than 5% of participants, 1 point for 2% to 5%, and 2 points for less than 2%).
